# Development of a Nanoparticle System for Controlled Release in Bioprinted Respiratory Scaffolds

**DOI:** 10.3390/jfb15010020

**Published:** 2024-01-12

**Authors:** Amanda Zimmerling, Christina Sunil, Yan Zhou, Xiongbiao Chen

**Affiliations:** 1 Division of Biomedical Engineering, College of Engineering, University of Saskatchewan, Saskatoon, SK S7N 5A9, Canadaxbc719@usask.ca (X.C.); 2 Vaccine and Infectious Disease Organization (VIDO), University of Saskatchewan, Saskatoon, SK S7N 5E3, Canada; yan.zhou@usask.ca; 3 Department of Mechanical Engineering, College of Engineering, University of Saskatchewan, Saskatoon, SK S7N 5A9, Canada

**Keywords:** nanoparticles, respiratory tissue engineering, hepatocyte growth factor, alginate, chitosan

## Abstract

The use of nanoparticle systems for the controlled release of growth factors is a promising approach to mimicking of the biochemical environment of native tissues in tissue engineering. However, sustaining growth factor release inside an appropriate therapeutic window is a challenge, particularly in bioprinted scaffolds. In this study, a chitosan-coated alginate-based nanoparticle system loaded with hepatocyte growth factor was developed and then incorporated into bioprinted scaffolds. The release kinetics were investigated with a focus on identifying the impact of the chitosan coating and culture conditions. Our results demonstrated that the chitosan coating decreased the release rate and lessened the initial burst release, while culturing in dynamic conditions had no significant impact compared to static conditions. The nanoparticles were then incorporated into bioinks at various concentrations, and scaffolds with a three-dimensional (3D) structure were bioprinted from the bioinks containing human pulmonary fibroblasts and bronchial epithelial cells to investigate the potential use of a controlled release system in respiratory tissue engineering. It was found that the bioink loaded with a concentration of 4 µg/mL of nanoparticles had better printability compared to other concentrations, while the mechanical stability of the scaffolds was maintained over a 14-day culture period. The examination of the incorporated cells demonstrated a high degree of viability and proliferation with visualization of the beginning of an epithelial barrier layer. Taken together, this study demonstrates that a chitosan-coated alginate-based nanoparticle system allows the sustained release of growth factors in bioprinted respiratory tissue scaffolds.

## 1. Introduction

Tissue engineering is a rapidly advancing field that aims to incorporate cells, biocompatible materials, and other bioactive molecules for the stimulation and control of tissue generation/regeneration. As stem cells and/or primary cells with differentiation potential are often utilized to generate more physiologically relevant engineered tissues, the cues and signaling factors provided by both the mechanical and chemical environment play a significant role in cellular development [1,2]. Biomechanical stimuli, such as mechanical loading, ventilation, or fluid movement/circulation, have all been shown to have an influence on cellular differentiation and growth, such as compressive loading leading to changes in cartilage formation [1,3,4]. The biochemical environment, including signaling molecules such as growth factors (GFs), also play a significant role in triggering and/or controlling cellular activities [5]. 

GFs, which are generally small glycoproteins naturally secreted and autoregulated by cells, have the capacity to activate sequential intracellular signaling pathways and influence gene expression. Many of the GFs and their applications in tissue engineering have already been summarized [6,7,8,9]. Hepatocyte growth factor (HGF) is one growth factor of interest as it has been shown to have anti-inflammatory and anti-apoptotic properties while also promoting angiogenesis and cell proliferation [10]. As a native biochemical component in the respiratory system, the addition of HGF has demonstrated the ability to promote healing and healthy epithelialization in injury models [10,11]. However, there are many challenges involved in the incorporation of GFs in tissue engineering application, including the sustaining of effective concentrations due to limited GF stability and/or short half-lives, sequential delivery, and the minimization of adverse effects [1,6,12,13]. Therefore, the methods for controlling the release of GFs within tissue-engineered constructs have become major areas of interest as they can maximize the time spent with the available concentration of the GF within its therapeutic window. 

Microparticles or nanoparticles are one of the means of aiding the controlled release of GFs, through encapsulation of the GF into a polymeric material before it is incorporated into an engineered construct or the tissue of interest. As the GF is then required to diffuse out of the particle or requires the degradation of the particle material in order to be released, the GF release rate can be slowed and/or sustained over an extended period [12,13,14,15]. While there are multiple methods for the fabrication of these micro- or nanoparticles, emulsion solvent evaporation techniques are still among the most common due to their simplicity and ability to create multilayered particles [16,17,18,19]. Emulsion techniques are also compatible with a wide range of materials, including the hydrogel materials commonly utilized in tissue engineering [16,17]. Alginate and chitosan are commonly utilized biomaterials that have previously been used to form microparticle systems [20,21,22,23,24,25]. For example, alginate, a water-soluble, biocompatible polysaccharide derived from brown algae, can be formed into GF-incorporating particles through the simple addition of GF and vigorous mixing with an oil and emulsifier solution [24]. As alginate microcarriers often undergo ionic crosslinking with CaCl_2_ in order to solidify, the crosslinking parameters (concentration, time, etc.) also play a role in release kinetics and cellular interactions by changing the porosity and surface profile of the formed particles [25,26]. The release kinetics and the surface profile can also be further modified through polymetric coatings. For example, chitosan, a natural non-toxic and biodegradable polymer found in crustacean shells and fungi, has been used to coat particles, to further slow and control GF release by limiting the original burst release, aiding in the maintenance of particle integrity over time and forming a secondary barrier to diffusion [24]. 

The application and characterization of these nanoparticle systems for controlled release can be further complicated by their inclusion into bioprinted scaffolds. Bioprinting, or the use of 3D printing technologies in concert with bioinks containing bioactive components, is a common technique utilized in tissue engineering due to its ability to create repeatable hierarchical structures that mimic physiological tissue [26,27,28]. The use of controlled release systems within 3D printed scaffolds has the capacity to further increase the relevance of the biochemical environment of the scaffolds [29]; however, the release kinetics of the particles may be influenced by the forces imposed on the particles during printing (for example, the shear forces imposed by extrusion through a needle), and diffusion through the scaffolding material may limit the bioactivity of the released growth factor [30,31,32]. Crosslinking of the biomaterial or hydrogel ink itself may also affect the release kinetics and the ability of loaded growth factors to diffuse through the scaffold, affecting the growth factor’s interactions with incorporated cells. Furthermore, the inclusion of controlled release systems within the bioink may affect the ink’s rheological properties, printability, and post-printing mechanical properties.

In this study, we hypothesized that the successful development of a controlled release system for HGF and its further incorporation into a bioprinted respiratory scaffold would have a beneficial effect on cellular proliferation and epithelialization within the scaffold. To test this, a chitosan-coated alginate nanoparticle system incorporating HGF was developed. Then, initial particle characterization occurred outside the scaffold system to elucidate the effects of the coating and culture conditions on the release. Upon the particles’ incorporation into the bioprinted respiratory system, an investigation into the morphology, bioink rheological properties, release kinetics, mechanical properties, and bioactivity was performed to elucidate the effects of the nanoparticle system on these parameters, which are important to the application of these systems in bioprinted respiratory scaffolds.

## 2. Materials and Methods

A summary table of all materials used in this study can be found in the supplementary materials (Table S1).

### 2.1. Cell Culture

Primary human bronchial epithelial cells (HBEpCs) (cryopreserved, C12640, LOT #468Z003) and primary human pulmonary fibroblasts (HPFs) (cryopreserved, C12360, LOT #446Z031), isolated from healthy human respiratory tissue, were purchased from PromoCell (Heidelberg, Germany). These cells were cultured in a standard incubator at 37 °C and 5% CO_2_ using Airway Epithelial Cell Growth Medium (C-21060, PromoCell), supplemented with Growth Medium Supplement Mix (C-39165, PromoCell), and Human Fibroblast Growth Medium 2 (C-23020, PromoCell), supplemented with Growth Medium 2 Supplemental Mix (C-39325), respectively. 1 × Anti-anti (Antiobiotic-antimyocotic, 15240-062, Gibco, Grand Island, NY, USA) was added to both culture conditions. The culture medium was changed every 2–3 days, with the cells split until confluent, until there were enough cells for printing.

### 2.2. Particle Preparation

Alginate microparticles were formed through the modification of a previously reported procedure [24]. Briefly, 5% alginate (low viscosity, A1112, Sigma-Aldrich, St. Louis, MO, USA) was dissolved in ultrapure water before 0.18% (wt.%) HGF (Recombinant human HGF protein (active), ab259401, Lot: GR3453764-1, Abcam, Cambridge, UK) was added to this solution and magnetically stirred for two hours. An oil phase was formed through mixing 1 mL of paraffin oil (18512, Sigma-Aldrich) with 100 µL Span 80 (viscosity 1000–2000 mPa·s, 85548, Sigma-Aldrich) for five minutes. Then, 300 µL of the loaded alginate solution was added dropwise to the oil phase while being magnetically stirred at 1200 rpm. After this emulsification had stirred for an hour, 150 µL of 0.68 M CaCl_2_ was added to the solution and mixed for an additional hour to crosslink the alginate particles. Then, 200 µL of isopropanol was added to the solution to harden the alginate spheres and stirred for 5 min. All the contents were then transferred to an Eppendorf tube with an additional 200 µL of isopropanol and centrifuged for 15 min. The separated liquid phase was then removed with a pipette. The collected nanoparticles were then washed three times with isopropanol and once with ultrapure water in order to remove any remaining oil phase. The particles were then resuspended and freeze-dried for 24 h.

In order to form the chitosan coating, 0.1% chitosan (448877, Sigma-Aldrich) was dissolved in 2% acetic acid before the pH was adjusted back to 5 through the addition of NaOH. Then, 200 µL of this chitosan solution, along with 100 µL of 5 mM CaCl_2_, was added to the particles and mixed on a rotary mixer for one hour. The particles were then centrifuged and washed with pure water three times before resuspending and freeze-drying overnight.

### 2.3. Scanning Electron Microscopy

Prepared particles were mounted on double-sided tape and gold-coated with a Quorum Q150TES Sputter Coater for scanning electron microscopy (SEM) using a Hitachi SU8010 SEM in order to assess particle morphology and size distribution. The samples were scanned at an accelerating voltage of 3.0 kV with magnifications ranging from 2000× to 50,000×.

### 2.4. Bioink Preparation 

A 3% alginate 0.075% collagen bioink was synthesized and used for all the experiments. Briefly, sodium alginate (180947, Sigma-Aldrich) was dissolved in Fibroblast Growth Medium 2 to form a 4% (*w*/*v*) solution. A 0.3% (*w*/*v*) collagen (type 1 bovine methacrylated collagen, PhotoCol, 9007-34-5) solution was formed through dissolution in acetic acid (pH = 5) before being added to the alginate solution in a 3:1 ratio. The pH of the final 3% alginate, 0.075% collagen solution was then adjusted back to neutral (pH 7–7.4) through the addition of NaOH. 

Depending on the study, various concentrations of prepared microparticles were mixed into the ink shortly before printing to determine the influence of the nanoparticle concentration.

### 2.5. Rheology

Rheological testing was carried out to determine the influence that the nanoparticle concentration had on the bioink fluid flow properties, with a specific focus on viscosity. This could then be further used to inform the selection of printing parameters, including print head speed and pressure. Shear-stress sweeps were carried out using a TA Instruments HR20 Discovery Series Rheometer fitted with 60.0 mm parallel plate geometry. The strain rate was varied from 0.1/s to 1000/s for microparticle concentrations ranging from 0 to 5 mg of nanoparticles/mL. The Cross model (Equation (1)) was fitted to the data using the Trios software (v5.6).

(1) $$\mu =\frac{\mu_{0}-\mu_{\infty }}{1+{\left(k\dot{\gamma }\right)}^{n}}+\mu_{\infty }$$

where *μ* is the dynamic viscosity, *μ_0_* is the zero-shear viscosity, *μ_∞_* is the infinite shear viscosity, *k* and *n* are fluid-specific parameters, and γ is the shear rate tensor. Based on the determined fluid specific parameters (*n* and *k*) from this fitting, the required pressure and speed for a desired flow rate can be calculated based on a selected needle diameter [33]. These settings can then be used as an initial starting point for printability analysis, although some changes to the calculated parameters may be required due to other variables, such as temperature, humidity, etc. 

### 2.6. Printability

Computer-aided design software was used to design a cylindrical scaffold with a diameter of 9 mm, height of 3 mm, and strand spacing of 1 mm, with the exception of the top layer, which was printed with a strand spacing of 0.27 mm, forming a solid surface layer. A GeSiM mbH BioScaffolder 3.2 outfitted with a 25-gauge needle was used to pneumatically extrude the bioink at a temperature of 37 °C into a six-well plate coated with 0.1% polyethyleneimine (PEI) and containing 50 mmol CaCl_2_. From previous experiments, the printability of the base bioink was assessed and the optimal printing speed (18 mm/s) and pressure (8 kPa) were determined [34]. Based on these previous studies, these parameters were tested to ensure consistency in the printing through imaging with a Leica AmScope Flexcam C1 microscope and image analysis with ImageJ 1.53 k after 20 min of immersion in the crosslinking media. 

### 2.7. Release Studies

A human HGF ELISA kit (Abcam, ab275901) was used to determine the concentration of HGF in the supernatant of the cultured constructs. Initial studies compared the release rate of the HGF that was directly incorporated into the alginate bioink as well as the HGF that was incorporated into the uncoated and coated nanoparticles. The following studies carried out throughout the culture of cell-containing constructs compared the release rates of HGF in dynamic and static culture conditions. An xMark microplate spectrophotometer was then used to measure absorbance at 450 nm.

### 2.8. Tensile Testing

Tensile testing was carried out with two goals in mind: to determine whether there was a difference between the mechanical stability of the scaffolds containing nanoparticles and that of those not containing nanoparticles and to determine whether the inclusion of 20 mmol CaCl_2_ in the culture media allowed the maintenance of the scaffold mechanical properties over the 14-day culture period. Briefly, cylindrical scaffolds (*n* = 6) 3 mm in height and 9 mm in diameter were printed using a GeSiM mbH BioScaffolder 3.2. All the bottom layers were printed with a strand spacing of 1 mm while the top layer was printed with a strand spacing of 0.27 mm in order to form a solid top layer. The scaffolds were incubated in a ventilated incubator with culture media containing 20 mmol CaCl_2_ and removed at the timepoints of 1, 3, 5, 7, 10, and 14 days. A tensile testing rate of 50 µm/second and a maximum displacement of 5 mm were used for testing due to the preliminary work identifying this crosshead speed as optimal for consistency in testing these soft hydrogel materials. The force–displacement data were converted into stress–strain curves for the analysis of Young’s modulus and the ultimate tensile strength. 

### 2.9. Bioprinting

Scaffolds containing 5 × 10^6^ HPFs/mL of bioink were bioprinted using the same 3 mm tall, 9 mm diameter design used in the tensile testing, with a lattice in the bottom layers to allow nutrient and waste transport between the cell-containing strands, while the top solid layers allowed effective seeding of the epithelial layer. HBEpCs were seeded on top of the cell-containing constructs at a density of 20,000 cells per construct. The bioprinted constructs were then cultured in a ventilated incubator in air–liquid interface conditions while the pressure and airflow were cycled to mimic the patterns of normal breathing at 37 °C. The scaffolds were removed for analysis at designated timepoints within a 28-day period. This design produces an air-exposed epithelial layer, supported by pulmonary fibroblasts in a hierarchical manner, increasing its relevance as a respiratory tissue scaffold.

### 2.10. Cellular Proliferation

Cell viability and proliferation were assessed on days 1, 3, 5, 7, 10, 14, 21, and 28. The scaffolds were removed from the culture, and the cell culture medium was removed. The scaffolds were washed in phosphate-buffered saline (PBS) before being dissolved in ethylenediaminetetraacetic acid tetrasodium salt dihydrate (EDTA) (E6511, Sigma). The cells were collected through centrifugation before being resuspended in PBS and transferred into a 96-well plate. WST-1 stain (Cell Proliferation Reagent WST-1, SKU5015944001, Sigma-Aldrich) was added to each well and incubated for an hour. Absorbance was then measured at 440 nm using an xMark microplate spectrophotometer.

### 2.11. Immunostaining

For confocal imaging at the timepoints of 1, 7, 14, 21, and 28 days, the cell culture medium was removed, and the scaffolds were washed with PBS before being fixed in 4% paraformaldehyde (PFA) (CAS: 50-00-0, Alfa Aesar, Haverhill, MA, USA). The scaffolds were then immersed in 30% sucrose for 1 h before being frozen in FSC 22 Clear Frozen Section Compound (3801480, Leica, Concord, ON, Canada) using isopentane (>99% purity, Thermo Scientific) and dry ice. The scaffolds were then stored in a −80 °C freezer before sectioning. Using an Avantik Biogroup cryostat (Avantik Biogroup LLC QS 11, Pine Brook, NJ, USA) at −20 °C, 20 µm sections were collected onto PEI-coated microscope slides. The slides were blocked for 2 h before primary antibodies, mouse monoclonal anti-vimentin (ab8069, Abcam, Cambridge, UK), and rabbit monoclonal anti-Pan-Cytokeratin (ab234297, Abcam), were added to the slides at a concentration of 2 µL/mL. After incubating at room temperature overnight, the slides were washed with PBS before secondary antibodies, Alexa-fluor 594 (A21207 IgG H+L, donkey anti-mouse, Life Technologies, Carlsbad, CA, USA), and Alexa-fluor 488 (A21202, IgG (H+L), donkey anti-rabbit) were added for 1 h. After washing, 4′,6′-diamidino-2-phenylindole (DAPI) (CAT#: 62248, Thermo Scientific) was added for 15 min. Coverslips were mounted using CitiFluor Mountant Solution (CAT. # 17970-25, Electron Microscopy Sciences, Hatfield, PA, USA) and left to dry protected from light. Preliminary imaging was carried out with a BioTek Lionheart LX automated microscope while confocal imaging was carried out using a Leica Microsystems confocal microscope (Leica Microsystems CMS GmbH TCS SP8). Image analysis and background removal were carried out when possible, using the Leica Microsystems software (v.5.0.2).

### 2.12. Statistical Analysis

All statistical analysis was carried out using Statistical Product and Service Solutions analysis software (SPSS 28). Non-parametric Kruskal-Wallis tests with pairwise comparisons with *p* < 0.05 were considered significant and were used for the analysis of the mechanical testing. General linear modelling with univariate analysis of variance was used for analysis of the release study and the cell viability analysis at a significance level of *p* < 0.05.

## 3. Results

### 3.1. SEM

**Figure 1:**
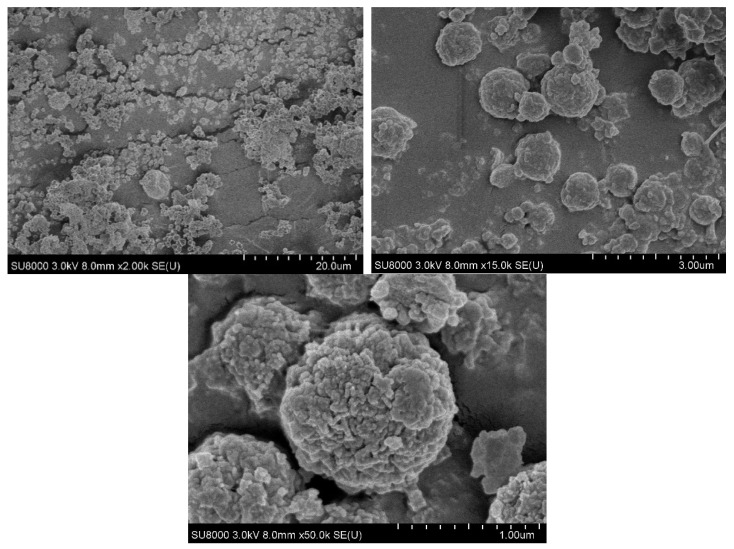
SEM images of chitosan-coated alginate nanoparticles at magnifications of 2000×, 5000×, and 50,000×.

As shown in Figure 1, consistent circular microparticles, with diameters averaging 940 ± 230 nm were obtained through the emulsion synthesis technique. 

At the lowest magnification of 2000×, dispersion of the particles on the mounting tape can be seen, with some visible agglomerations of particles. However, as the magnification is increased to 5000× and 50,000×, individual particles with a distribution of diameters from 600 nm to 1000 nm are visible. Furthermore, the 50,000× magnification makes it evident that the nanoparticles have a rough exterior, with an increased surface area.

### 3.2. Rheology

**Figure 2:**
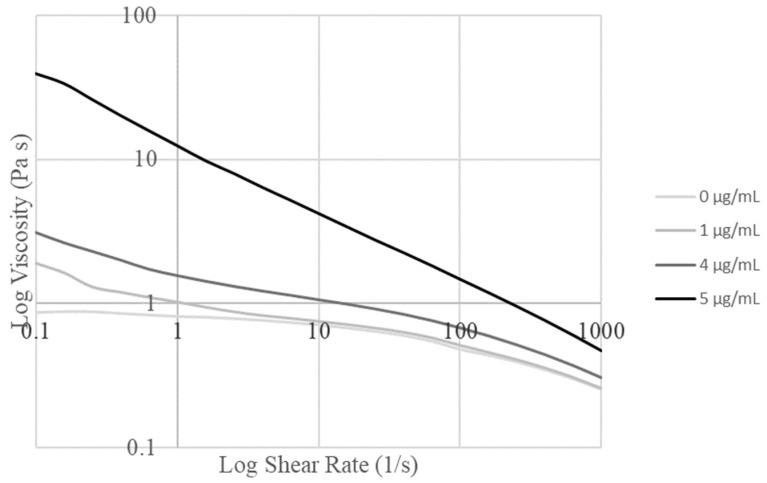
Logarithmic graph of viscosity versus shear rate for various concentrations of nanoparticles in the base alginate collagen bioink.

Shear stress sweeps from 0.1/s to 1000/s were carried out and graphed for the alginate/collagen bioink with various nanoparticle concentrations (Figure 2). Concentrations of 1 µg/mL and 5 µg/mL were tested first, before moderate concentrations were tested to determine the maximum concentration of particles that could be loaded before the bioink properties were changed to the extent that the print settings would require variation.

Based on the obtained graphs, the Trios software determined the Cross model as the best fit for all the obtained curves. The parameter values determined by this fit are summarized in Table 1. The increase in viscosity seen with increased nanoparticle concentration in Figure 2 can be seen to correspond to the increase in zero-rate viscosity with the nanoparticle concentration in Table 1. The optimal concentration was determined to be 4 µg/mL due to the large change in fluid flow parameters identified in the fit model between 4 µg/mL and 5 µg/mL (Table 1), which corresponded to the requirement to change print settings to maintain printability.

### 3.3. Printability

**Figure 3:**
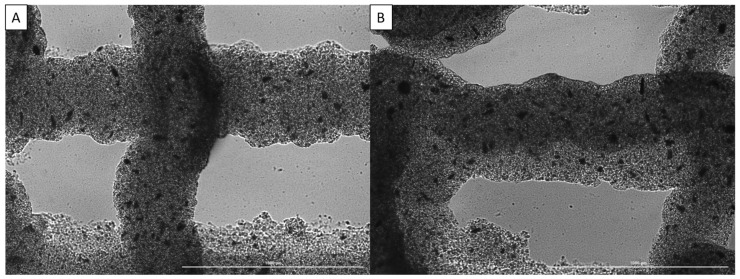
Imaging of strand dimensions for alginate/collagen bioink with various nanoparticle concentrations: (**A**) 0 µg/mL, (**B**) 4 µg/mL. Scale bar of 1000 µm.

Printability was assessed through printing two-layer scaffolds using pre-determined parameters for the base bioink, imaging the scaffolds, and then measuring dimensions such as strand diameter to determine how closely it matched the set CAD design, as previously described [35]. Some of the images used for assessment can be seen in Figure 3. 

From these images, it can be seen that increasing the nanoparticle concentration from 0 µg/mL to 4 µg/mL results in less drooping of the upper layer strands between the gaps in the bottom layer strands, while the strand diameter is slightly decreased. This resulted in an increase in printability through the addition of nanoparticles to the bioink; however, in some prints, clogging became an issue as the nanoparticles agglomerated. This was prevented as much as possible through sifting the nanoparticles before adding them to the biomaterial. 

### 3.4. Release Studies

**Figure 4:**
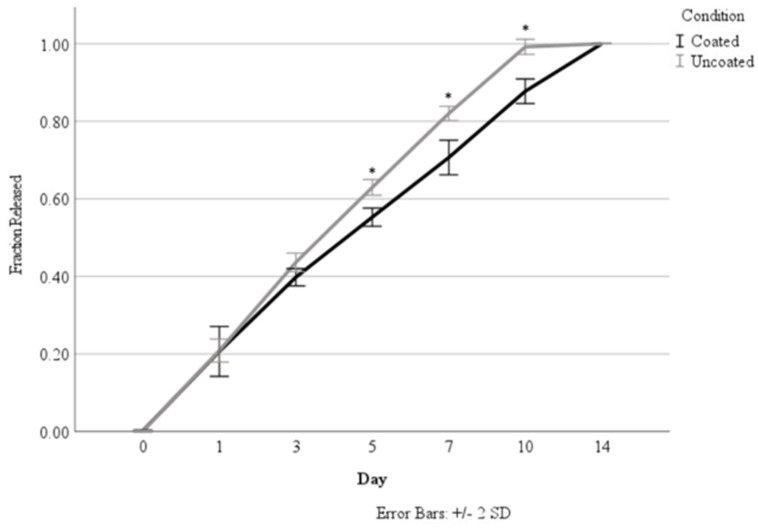
Comparison of HGF release from coated versus uncoated nanoparticles. Asterisks (*) indicate a significant difference (*p* < 0.05).

Originally, the release studies were carried out over a 14-day time period to determine the effect of coating the alginate nanoparticles in chitosan based on a calculated loading concentration of 7.28 ng/mL. As seen in Figure 4 and Table 2 and Table 3, the chitosan coating demonstrated a significant reduction in the total percentage of HGF released, starting at Day 5. The coating was able to sustain release over 14 days, whereas the uncoated nanoparticles had released all of their incorporated HGF by Day 10. Nanoparticles with and without coating were able to demonstrate sustained release over time.

**Figure 5:**
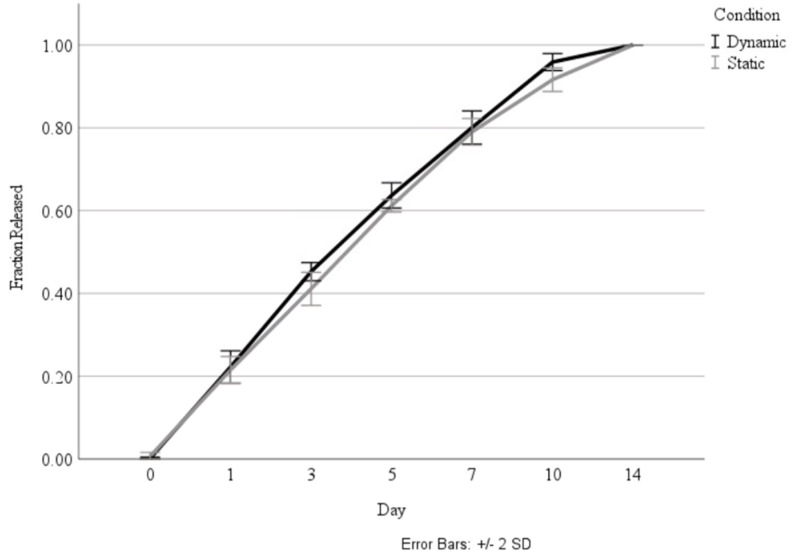
Comparison of total fraction of HGF release over 14 days during culture in static and dynamic conditions.

Following the investigation into the effect of the coating, the coated nanoparticles were cultured in both standard incubation conditions, as well as in a bioreactor that mimicked the pressure changes and airflow that occur in the lung, to determine whether the biomechanical stimulus would influence the release rate. As shown in Figure 5, there was no significant difference in the release rate between the static and dynamic culture conditions.

As seen in Table 2 and Table 3, the release rate of all the systems was stabilized between Day 3 and Day 7, and a release plateau around 9%/day was reached. The burst release in the first 24 h is evident, while the concentration released past 7 days begins to taper off significantly. 

### 3.5. Tensile Testing

**Figure 6:**
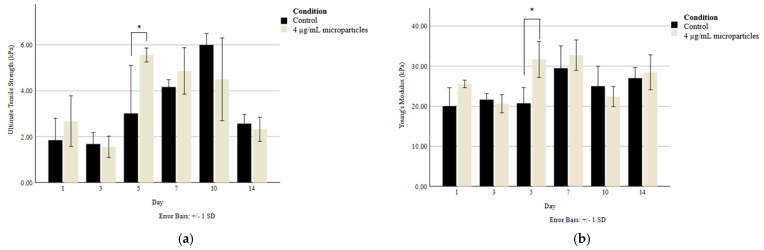
(**a**) Ultimate tensile strength and (**b**) Young’s modulus of 3D printed scaffolds without and with 4 µg/mL nanoparticles. Asterisks (*) indicate a significant difference (*p* < 0.05) between conditions at the same timepoint.

Tensile testing was carried out on 3D printed scaffolds to determine whether there was any difference in the Young’s modulus and ultimate tensile strength (UTS) between the scaffolds without nanoparticles and those with 4 µg/mL of nanoparticles. With the goal of maintaining the scaffolds’ structural integrity over the 14-day culture period, the scaffolds were cultured in media containing 20 mmol CaCl_2_, which allowed the crosslinking of the alginate-based bioink to be maintained without a reduction in cellular compatibility. As seen in Figure 6, there was only a significant difference in tensile strength between the control and the nanoparticle-containing scaffolds on Day 5. Generally, the Young’s modulus was maintained at a consistent range of 20–30 kPa, while the UTS varied to a greater extent but was generally sustained between 2 and 6 kPa through the addition of CaCl_2_ to the culture media.

### 3.6. Cellular Proliferation

**Figure 7:**
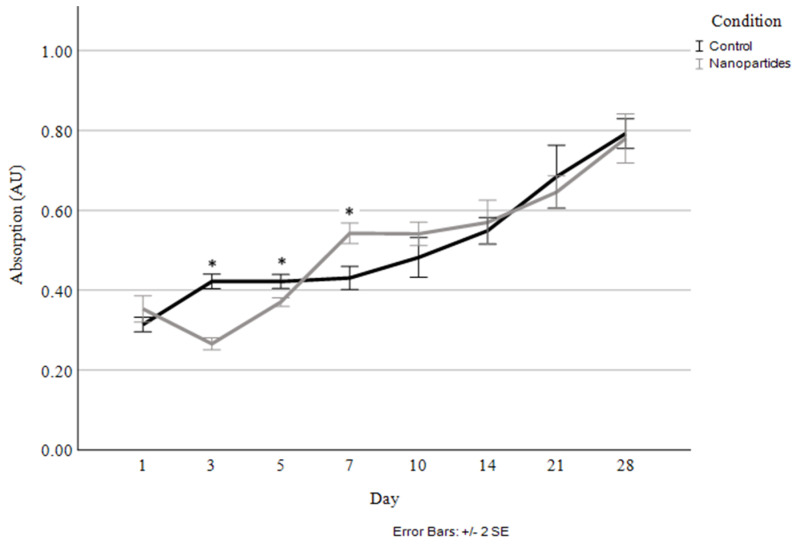
Cellular proliferation and viability quantified through live cell staining and absorbance measurement for control and 4 µg/mL HGF-loaded nanoparticles. Asterisks (*) indicate a significant difference (*p* < 0.05).

**Figure 8:**
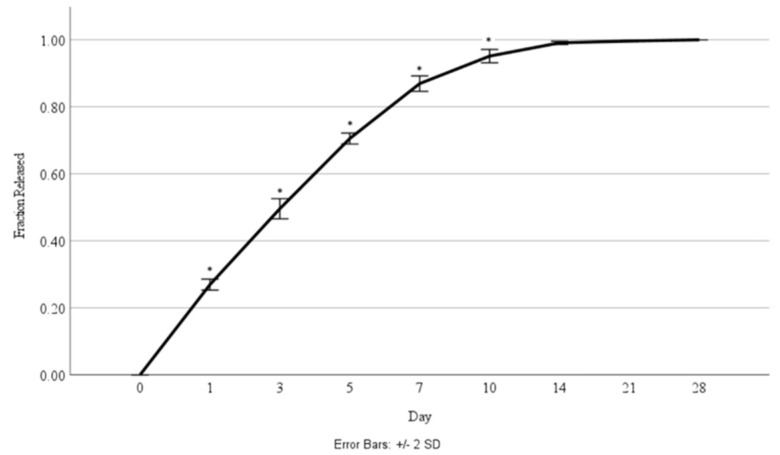
Fraction of HGF released over a 28-day time period during culture with cells. Asterisks (*) indicate a significant difference (*p* < 0.05).

Cellular proliferation was compared between the scaffolds without nanoparticles and those with 4 µg/mL of HGF-loaded nanoparticles (Figure 7). The corresponding percentage of HGF released was also measured at each timepoint (Figure 8).

As seen in Figure 7, the only significant differences in cellular proliferation between the control and the nanoparticle-containing scaffolds occurred at the early time points of days 3, 5, and 7. Originally, at days 3 and 5, the nanoparticle-containing scaffolds exhibited lesser cell proliferation and a decrease in live cells in comparison to Day 1; however, shortly after that, the cells begin proliferating rapidly in the nanoparticle scaffolds, resulting in them having a greater number of viable cells at Day 7 than the control. At this point, cellular proliferation remained highly consistent between the control and nanoparticle-containing scaffolds, with a steady increase in the cell numbers in the scaffolds seen up to the 28-day timepoint. 

### 3.7. Immunostaining

**Figure 9:**
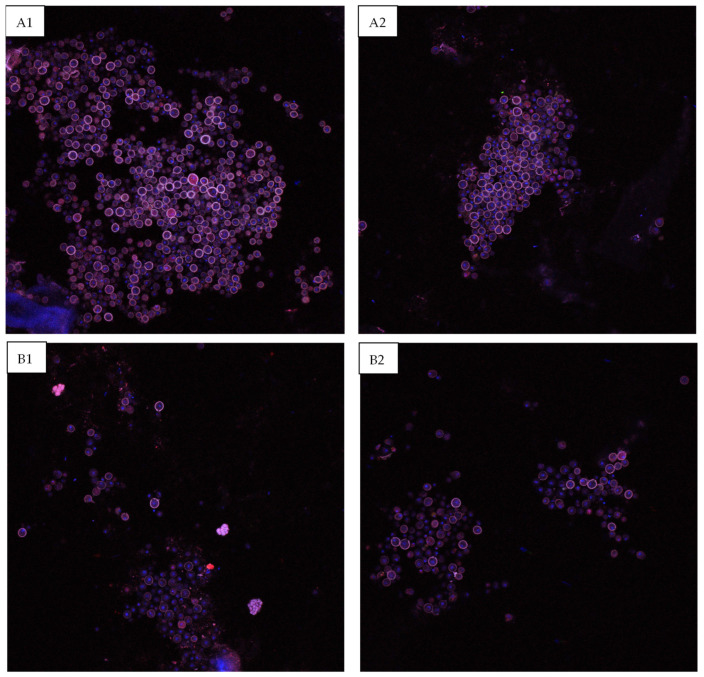
Confocal images of the epithelial surface layer of the bioprinted constructs at the Day 14 timepoint. (**A1**,**A2**) Scaffold containing 4 µg/mL HGF-loaded nanoparticles, (**B1**,**B2**) control.

The scaffolds were sectioned in order to allow imaging of the surface epithelial surface to determine whether the HGF-containing nanoparticles stimulated increased epithelial cell proliferation and differentiation. As can be seen in Figure 9, at the Day 14 timepoint, the scaffolds that contained nanoparticles exhibited a higher degree of Pac-CK stained epithelial cells on the barrier surface. 

## 4. Discussion

This study demonstrates a successful method for the synthesis of growth factor-loaded chitosan-coated alginate nanoparticles with dimensions ranging from 600 to 1000 nm and their inclusion into a bioprinted respiratory scaffold. Based on previous studies utilizing a similar production technique [13,24], the emulsion technique with a set stir speed allows consistent and repeatable nanoparticle synthesis within a similar size range (0.8–2.3 µm or 700–900 nm, respectively), demonstrating consistency in the production method. In order to be able to utilize the knowledge gained through a previous bioprinting study [30], rheological analysis was carried out to determine what concentration of nanoparticles could be added to the bioink without having a large impact on the fluid properties in order to maintain a high degree of printability and to allow comparison with previous works. Originally, 1 µg/mL and 5 µg/mL concentrations were tested. The concentration was then stepped down from 5 µg/mL (4, 3, 2 µg/mL), until it was determined that the largest change in properties occurred between 4 and 5 µg/mL. Due to this, 4 µg/mL was selected as the optimal concentration to load into the bioink to have maximal growth factor included without negatively impacting printability. At this concentration, the inclusion of nanoparticles was seen to maintain printability without adjusting the print parameters from an unloaded bioink, although clogging due to particle agglomeration was a rare occurrence.

A direct comparison of the release rate with the results reported in the literature is impossible due to differences in preparation, culture conditions, and loaded molecules between the studies [13,24,36,37,38]; however, the general trends remain consistent. As seen in this study, the addition of the chitosan coating on the alginate microparticles demonstrated an increased retention of the loaded molecule and slightly decreased the initial burst release. However, the HGF-loaded nanoparticles seem to extend the release of HGF for a longer time period than that reported in other studies [24]. It has previously been reported in the literature that increased alginate concentration and a longer drying time of 24 h may contribute to this elongated release period [13]. Furthermore, as these nanoparticles were incorporated into a scaffolding material, the degradation of the particles and diffusion of the loaded HGF may have been slowed by the higher viscosity of the matrix material. This longer release time when HGF is incorporated into a nanoparticle system, which is then further incorporated into a bioprinted system, indicates that combinatorial techniques may be very useful in sustaining concentrations of growth factors within certain concentrations over a longer time frame based on the slowed diffusion and particle degradation. 

With the goal of maintaining consistent mechanical properties over the entire culture period, the nanoparticle-containing and control alginate/collagen scaffolds were cultured in media containing 20 mmol CaCl_2_. This was conducted as previous studies have demonstrated that these scaffolds undergo significant loss of mechanical properties over 14 days of culture, leading to the possible loss of cells and cell hierarchy within the structure [34]. In comparison to that study, which did not include any crosslinker in the culture media, this study was able to maintain relatively consistent mechanical properties over the culture period, indicating the ability to use these conditions for longer term cultures, as reported in the literature [39]. The ability to maintain consistent mechanical properties over a long-term culture period helps to increase the biomimicry of the system, as the matrix material can be tailored to have mechanical properties similar to those of the system of interest, in this case the bronchioles of the human respiratory tract. In this study, the maintenance of the mechanical stability of the nanoparticle-incorporating constructs also likely contributed to the elongated release period, both in static and dynamic conditions as the inclusion of crosslinker in the culture media likely helped to maintain particle integrity along with scaffold integrity, causing the maintenance of the diffusion barrier.

As alginate nano- and microparticles are known to exhibit a burst release of their loaded molecules at the early stages of culture, which is seen to some degree in the same percentage of release being seen over one day (Day 1) and two days later on Day 3 (Table 2), it is possible that the high concentration of release was a cause of the decreased cellular proliferation on Day 3 for the nanoparticle-containing scaffolds. This also may be due to the cell viability being decreased when nanoparticles are included in an extruded bioink, due to the application of additional shear forces during extrusion; however, between Day 3 and Day 7, the nanoparticle-containing scaffolds demonstrate consistent growth factor release and greater cell viability, indicating good cellular biocompatibility once the initial burst release and shear stresses have been overcome. While differences between the live cell numbers within the control and nanoparticle-containing scaffolds are insignificant at all later timepoints, confocal imaging of the Day 14 scaffolds indicates that a more coherent epithelial surface layer was formed in the HGF-containing scaffolds; however, this is difficult to quantify through imaging. 

In physiological systems, HGF is constantly present but is rapidly upregulated in the case of injury to help stimulate epithelial cell proliferation and wound healing [40,41]. After pneumonectomy in mice, HGF is seen to be upregulated in both the alveoli and the airways with concentrations in the lung increasing to up to 140 ng/g of tissue up to 10 days post-surgery, while the control group HGF concentration was maintained at around 90 ng/g of tissue [40]. More recent investigation of alveolar wound healing was carried out on a chip system, and it was determined that a concentration of 10 ng/mL of culture media markedly accelerated wound healing, while increasing concentrations past 10 ng/mL only caused slight further improvement [41]. Based on the release kinetics seen in this study (Figure 7), with approximately 30% of the total loaded growth factor being released by Day 1 (2.2 ng/mL of bioink), followed by an approximate 20% release by Day 3, Day 5, and Day 7 (1.44 ng/mL of bioink), it would be expected that an increased concentration of HGF may cause a greater effect; however, increasing this loaded concentration is challenging due to the impact of nanoparticle inclusion in the bioink on printability and the limits to the volume of growth factor that efficiently loads into the nanoparticle systems. It would be interesting for future studies to investigate direct loading of the growth factor into the bioink to stimulate an initial injury-like growth factor response, while also including a controlled release system to sustain a level of the growth factor within the bioprinted constructs over a longer time period. Together, both would allow the increasing of the weight percent of growth factor originally loaded into the system. 

## 5. Conclusions

Controlled release systems that consist of nanoparticles incorporating signaling molecules can be used to help sustain the release and presence of biochemical stimuli to mimic the biochemical environment of native tissues. This study aimed to develop a chitosan-coated alginate nanoparticle system and to investigate its ability to sustain the release of HGF within a 3D bioprinted respiratory tissue construct. The results illustrated that synthesis of chitosan-coated alginate nanoparticles through an emulsion technique resulted in spherical nanoparticles with an average diameter of 900 ± 230 nm. The chitosan coating was able to sustain the release of HGF over a 14-day period. While the addition of nanoparticles increased the viscosity of the alginate/collagen bioink, a concentration of 4 µg/mL was found to be the best in terms of printability at the set printing parameters. The mechanical properties of the scaffolds were maintained through the addition of crosslinker in the culture media, allowing cellular studies to take place over 28 days without significant degradation of the scaffolds. The cell viability was maintained, and the epithelial cell barrier formation was visualized via confocal microscopy. Taken together, this study demonstrates the successful synthesis and application of a controlled release system of growth factors in tissue-engineered respiratory tissue scaffolds.

## Figures and Tables

**Table 1:** Fit parameters and R^2^ value for the Cross model on material with varied nanoparticle concentrations.

Nanoparticle Concentration (µg/mL)	Zero-Rate Viscosity (Pa·s)	Infinite-Rate Viscosity (Pa·s)	*n*	*k*	R^2^
0	0.90	1 × 10^−13^	0.71	1.89	1.00
1	2.63	2 × 10^−13^	0.70	2.10	0.98
4	4.95	1 × 10^−12^	0.69	2.76	0.99
5	142406	3 × 10^−2^	0.51	14.08	1.00

**Table 2:** Percent of total loaded HGF release per day over a 14-day period.

	Day 0	Day 1	Day 3	Day 5	Day 7	Day 10	Day 14
Static	0	22%	10%	10%	10%	4%	2%
Dynamic	0	23%	12%	9%	8%	5%	1%
Coated	0	22%	9%	8%	8%	5%	3%
Uncoated	0	21%	11%	10%	9%	6%	0%

**Table 3:** Corresponding growth factor release per day (ng/mL).

	Day 0	Day 1	Day 3	Day 5	Day 7	Day 10	Day 14
Static	0.00	1.59	0.71	0.70	0.70	0.29	0.15
Dynamic	0.00	1.64	0.84	0.66	0.59	0.37	0.08
Coated	0.00	1.59	0.64	0.60	0.59	0.39	0.22
Uncoated	0.00	1.56	0.78	0.73	0.69	0.43	0.01

## Data Availability

All data collected and analyzed in this manuscript are available upon reasonable request from the corresponding author.

## Supplementary Materials

The following supporting information can be downloaded at: https://www.mdpi.com/article/10.3390/jfb15010020/s1, Table S1: Materials used in the nanoparticles study.

## References

[1] Tian B., Lin W., Zhang Y. (2021). Effects of biomechanical forces on the biological behaviour of cancer stem cells. J. Cancer.

[2] Li J., Li Y., Zhang Y., Enhejirigala B., Li Z., Song W., Wang Y., Duan X., Yuan X., Fu X. (2021). Biophysical and Biochemical Cues of Biomaterials Guide Mesenchymal Stem Cell Behaviours. Front. Cell Dev. Biol..

[3] Huh D., Leslie D., Matthews B., Fraser J., Jurek S., Hamilton G., Thornlow K., McAlexander M., Ingber D. (2012). A Human Disease Model of Drug Toxicity-Induced Pulmonary Edema in a Lung-on-a-Chip Microdevice. Sci. Transl. Med..

[4] Sardroud H., Chen X., Eames F. (2023). Applied Compressive Strain Governs Hyaline-Like Cartilage Versus Fibrocartilage-Like ECM Produced Within Hydrogel Constructs. Int. J. Mol. Sci..

[5] Aguilar L., Silva S., Mouton S. (2019). Growth factor delivery: Defining the next generation platforms for tissue engineering. J. Contr. Release.

[6] Tsiklin I., Shabunin A., Kolsanov A., Volova L. (2022). In Vivo Bone Tissue Engineering Strategies: Advances and Prospects. Polymers.

[7] Agha E., Seeger W., Bellusci S. (2016). Therapeutic and pathological roles of fibroblast growth factors in pulmonary diseases. Dev. Dyn..

[8] O’Leary C., Gilbert J., O’Dea S., O’Brien F., Cryan S. (2015). Respiratory Tissue Engineering: Current Status and Opportunities for the Future. Tissue Eng.-Part B Rev..

[9] Bao H., Huang J., Zhang Z. (2022). Functional Au nanoparticles for engineering and long-term CT imaging tracking of mesenchymal stem cells in idiopathic pulmonary fibrosis treatment. Biomaterials.

[10] Panganiban R., Day R. (2010). Hepatocyte growth factor in lung repair and pulmonary fibrosis. Acta Pharmacol. Sin..

[11] Wang H., Yang Y., Zhao L., Xiao F., Zhang Q., Wen M., Wu C., Peng R., Wang L. (2013). Hepatocyte growth factor gene-modified mesenchymal stem cells reduce radiation-induced lung injury. Hum. Gene Ther..

[12] Subbiah R., Guldberg R. (2018). Materials Science and Design Principles of Growth Factor Delivery Systems in Tissue Engineering and Regenerative Medicine. Adv. Healthc. Mater..

[13] Yasmin F., Chen X., Eames F. (2019). Effect of Process Parameters on the Initial Burst Release of Protein-Loaded Alginate Nanospheres. J. Funct. Biomater..

[14] El-ezz D., Abdel-Rahman L., Al-Fahran B., Mostafa D., Ayad E., Basha M., Abdelaziz M., Abdalla E. (2022). Enhanced In Vivo Wound Healing Efficacy of a Novel Hydrogel Loaded with Copper (II) Schiff Base Quinoline Complex (CuSQ) Solid Lipid Nanoparticles. Pharmaceuticals.

[15] Begines B., Ortiz T., Perez-Aranda M., Martinez G., Merinero M., Arguelles-Arias F., Alcudia A. (2020). Polymeric Nanoparticles for Drug Delivery: Recent Developments and Future Prospects. Nanomaterials.

[16] Jenjob R., Phakkeeree T., Seidi F., Theeraslip M., Crespy D. (2019). Emulsion Techniques for the Production of Pharmacological Nanoparticles. Macromol. Biosci..

[17] Gharieh A., Khoee S., Mahdavian A. (2019). Emulsion and mini emulsion techniques in preparation of polymer nanoparticles with versatile characteristics. Adv. Colloid. Interface Sci..

[18] Bendre A., Bhat M., Lee K., Altalhi T., Alruqi M., Kurkuri M. (2022). Recent developments in microfluidic technology for synthesis and toxicity-efficiency studies of biomedical nanomaterials. Mater. Today Adv..

[19] Kulkarni M., Goel S. (2020). Microfluidic devices for synthesizing nanomaterials—A review. Nano Express.

[20] Sohail R., Abbas S. (2020). Evaluation of amygdalin-loaded alginate-chitosan nanoparticles as biocompatible drug delivery carriers for anticancerous efficacy. Int. J. Biol. Macromol..

[21] Taghiloo S., Ghajari G., Zand Z., Kabiri-Samani S., Kabiri H., Rajaei N., Piri-Gharaghie T. (2023). Designing Alginate/Chitosan Nanoparticles Containing *Echinacea angustifolia*: A Novel Candidate for Combating Multidrug-Resistant *Staphylococcus aureus*. Chem. Biodivers..

[22] Kaur J., Kour A., Panda J., Harjai K., Chhibber S. (2020). Exploring Endolysin-Loaded Alginate-Chitosan Nanoparticles as Future Remedy for Staphylococcal Infections. AAPS PharmSciTech.

[23] Sun L., Nie X., Lu W., Zhang Q., Fang W., Gao S., Chen S., Hu R. (2022). Mucus-Penetrating Alginate-Chitosan Nanoparticles Loaded with Berberine Hydrochloride for Oral Delivery to the Inflammation Site of Ulcerative Colitis. AAPS PharmSciTech.

[24] Zhai P., Chen X., Schreyer D. (2013). Preparation and characterization of alginate microspheres for sustained protein delivery within tissue scaffolds. Biofabrication.

[25] Pan C., Chien S., Chiang T., Yen C., Wang S., Wen Z., Yu C., Shiue Y. (2020). Optimization for the spherical integrity for sustained-release alginate microcarriers- encapsulated doxorubicin by the Taguchi method. Sci. Rep..

[26] Amirian J., Tripathi G., Kang H., Lee B. (2022). Porous BMP-2 immobilized PLGA/Glycol chitosan scaffold with enhanced hydrophilicity, mineralization and osteogenesis. Mater. Lett. Part B.

[27] Saini G., Segaran N., Mayer J., Saini A., Albadawi H., Oklu R. (2021). Applications of 3D Bioprinting in Tissue Engineering and Regenerative Medicine. J. Clin. Med..

[28] Kakarla A., Kong I., Turek I., Kong C., Irving H. (2022). Printable gelatin, alginate and boron nitride nanotubes hydrogel-based ink for 3D bioprinting and tissue engineering applications. Mater. Des..

[29] Yang J., Li Z., Li S., Zhang Q., Zhou X., He C. (2023). Tunable metacrylated silk fibroin-based hybrid bioinks for the bioprinting of tissue engineerin scaffolds. Biomater. Sci..

[30] Izudak B., Bal-Ozturk A. (2022). The effect of LDHs nanoparticles on the cellular behavior of stem cell-laden 3D-bioprinted scaffold. J. Biomater. Appl..

[31] Aghajanpour S., Esfandyari-Manesh M., Ghahri T., Ghahremani M., Atyabi F., Heydari M., Motasadizadeh H., Dinarvand R. (2022). Impact of oxygen-calcium-generating and bone morphogenetic protein-2 nanoparticles on survival and differentiation of bone marrow-derived mesenchymal stem cells in the 3D bio-printed scaffold. Colloids Surf. B Biointerfaces.

[32] Marovic N., Ban I., Maver U., Maver T. (2023). Magnetic nanoparticles in 3D-printed scaffolds for biomedical applications. Nanotechnol. Rev..

[33] Mendieta J., Fontanarosa D., Wang J., Paritala P., McGahan T., Lloyd T., Li Z. (2020). The importance of blood rheology in patient-specific computational fluid dynamics simulation of stenotic carotid arteries. Biomech. Model. Mechanobiol..

[34] Zimmerling A., Boire J., Zhou Y., Chen X. (2023). Influence of Biomechanical Stimulus on 3D Bioprinted Respiratory Tissue Scaffolds. Preprint.

[35] Zimmerling A., Yazdanpanah Z., Cooper D., Johnston J., Chen X. (2021). 3D printing PCL/nHA bone scaffold: Exploring the influence of material synthesis techniques. Biomater. Res..

[36] Ribeiro A., Neufeld R., Arnaud P., Chaumeil J. (1999). Microencapsulation of lipophilic drugs in chitosan-coated alginate microspheres. Int. J. Pharm..

[37] Yang D., Gao K., Bai Y., Lei L., Jia T., Yang K., Xue C. (2021). Microfluidic synthesis of chitosan-coated magnetic alginate microparticles for controlled and sustained drug delivery. Int. J. Biol. Macromol..

[38] Yousefi M., Khanniri E., Shadnoush M., Khorshidian N., Mortazavian A. (2020). Development, characterization and in vitro antioxidant activity of chitosan-coated alginate microcapsules entrapping *Viola odorata* Linn. extract. Int. J. Biol. Macromol..

[39] Berg J., Weber Z., Fechler-Bitteti M., Hocke A., Hippenstiel S., Elomaa L., Weinhart M., Kurreck J. (2021). Bioprinted Multi-Cell Type Lung Model of the Study of Viral Inhibitors. Viruses.

[40] Sakamaki Y., Matsumoto K., Mizuno S., Miyoshi S., Matsuda H., Nakamura T. (2002). Hepatocyte Growth Factor Stimulates Proliferation of Respiratory Epithelial Cells during Postpneumonectomy Compensatory Lung Growth in Mice. Am. J. Respir. Cell Mol. Biol..

[41] Felder M., Trueeb B., Stucki A., Borcard S., Stucki J., Schnyder B., Geiser T., Guenat O. (2019). Impaired Wound Healing of Alveolar Lung Epithelial Cells in a Breathing Lung-On-A-Chip. Front. Bioeng. Biotechnol..

