# Supplementary material for: Development of a Nanoparticle System for Controlled Release in Bioprinted Respiratory Scaffolds
Source: J Funct Biomater. 2024 Jan 12;15(1):20. doi: 10.3390/jfb15010020 (PMC10816437; doi:10.3390/jfb15010020)
Supplement: Supplementary file 1 [file jfb-15-00020-s001.zip › jfb-2746848-supplementary.pdf]

Journal of Functional Biomaterials

Supplementary Material

Development of a Nanoparticle System for Controlled Release in Bioprinted Respiratory Scaffolds

Amanda Zimmerling [1,2,*], Christina Sunil [1], Yan Zhou [2] and Xiongbiao Chen [1,3]

[1] Division of Biomedical Engineering, College of Engineering, University of Saskatchewan, Saskatoon, SK S7N 5A9, Canada; ikl420@usask.ca (C.S.); xbc719@usask.ca (X.C.)
[2] Vaccine and Infectious Disease Organization, (VIDO), University of Saskatchewan, Saskatoon, SK S7N 5E3, Canada; yan.zhou@usask.ca
[3] Department of Mechanical Engineering, College of Engineering, University of Saskatchewan, Saskatoon, SK S7N 5A9, Canada
* Correspondence: asz694@usask.ca

Citation: Zimmerling, A.; Sunil, C.; Zhou, Y.; Chen, X. Development of a Nanoparticle System for Controlled Release in Bioprinted Respiratory Scaffolds. *J. Funct. Biomater.* **2024**, *15*, 20. https://doi.org/10.3390/jfb15010020

Academic Editor: Nenad Ignjatovic

Received: 14 November 2023
Revised: 21 December 2023
Accepted: 5 January 2024
Published: 12 January 2024

Copyright: © 2024 by the authors. Licensee MDPI, Basel, Switzerland. This article is an open access article distributed under the terms and conditions of the Creative Commons Attribution (CC BY) license (https://creativecommons.org/licenses/by/4.0/).

Table S1. Materials used in the nanoparticles study.

Section	Notes	Catalogue Number	Lot #	Supplier
**Cell Culture**				
**Primary Human Bronchial Epithelial Cells**	cryopreserved	C12640	468Z003	Promocell
**Primary Human Lung Fibroblasts**	cryopreserved	C12360	446Z031	Promocell
**Airway Epithelial Cell Growth Medium**		C21060		Promocell
**Growth Medium Supplement Mix**		C39265		Promocell
**Human Fibroblast Growth Medium-2**		C23020		Promocell
**Growth Medium 2 Supplemental Mix**		C39325		Promocell
**Anti-Anti**		15240-062		Gibco
**Nanoparticle Preparation**				
**Sodium Alginate**	low viscosity	A1112		Sigma-Aldrich
**Hepatocyte Growth Factor**		ab259401	GR3453764-1	abcam
**Paraffin Oil**		18512		Sigma-Aldrich
**Span 80**	viscosity 1000-2000 mPa.s	85548		Sigma-Aldrich
**$CaCl_2$**		223506		Sigma-Aldrich
**Isopropanol**		I9030	29996TK	Sigma-Aldrich
**Chitosan**		448877		Sigma-Aldrich
**NaOH**		1003614357		Sigma-Aldrich
**Bioink Preparation**				
**Sodium Alginate**	medium viscosity	180947		Sigma-Aldrich
**Human Fibroblast Growth Medium-2**		C23020		Promocell
**Collagen**	type 1 bovine methacrylated	9007-34-5		PhotoCol
**Acetic Acid**		351271	522112	Fisher
**NaOH**		1003614357		Sigma-Aldrich
**Bioprinting**				
**Polyethyleneimine**		M26B040	J61270	Alfa Aesar
**Release Studies**				
**Human HGF ELISA Kit**		ab275901		abcam
**Cell Viability and Proliferation**				
**Phosphate Buffered Saline**	10x concentrate	P5493	SLBX1610	Sigma
**Ethylenediaminetetraacetic Acid Tetrasodium Salt Dihydrate**		E6511		Sigma
**WST-1**		SKU5015944001		Sigma-Aldrich
**Immunostaining**				
**Paraformaldehyde**		CAS:50-00-0		Alfa Aesar
**Sucrose**	>99.5%	S0389	SLCJ6927	Sigma
**FSC 22 Clear Frozen Section Compoud**		3801480		Leica
**Isopentane**	>99% purity	78-78-4		ThermoScientific

**Mouse Monoclonal Anit-Vimentin**		ab8069	abcam
**Rabbit Monoclonal Anit-Pan-Cytokeratin**		ab234297	abcam
**Alexa Fluor 595**	IgG H+L, donkey- anti mouse	A21207	Life Technologies
**Alexa Fluor 488**	IgG H+L, donkey- anti rabbit	A21202	Life Technologies
**DAPI**		62248	ThermoScientific
**CitiFluor Mountant Solution**		17970-25	Electron Microscopy Sciences
